# Preventing *Staphylococci* Surgical Site Infections with a Nitric Oxide-Releasing Poly(lactic acid-*co*-glycolic acid) Suture Material

**DOI:** 10.1021/acsabm.4c00128

**Published:** 2024-04-23

**Authors:** Lauren Griffin, Mark Richard Stephen Garren, Patrick Maffe, Sama Ghalei, Elizabeth J. Brisbois, Hitesh Handa

**Affiliations:** † School of Chemical, Materials and Biomedical Engineering, College of Engineering, University of Georgia, Athens, Georgia 30602, United States; ‡ Department of Pharmaceutical and Biomedical Sciences, College of Pharmacy, University of Georgia, Athens, Georgia 30602, United States

**Keywords:** nitric oxide, surgical site infection, suture, *Staphylococci*, antibacterial

## Abstract

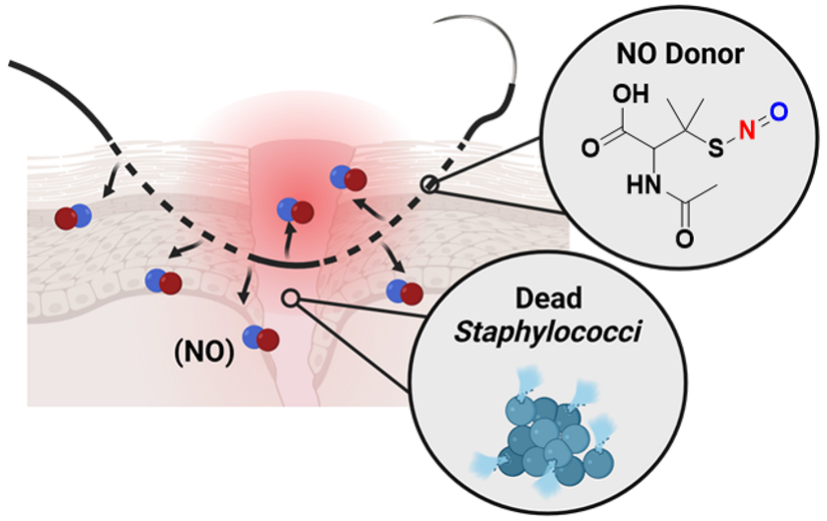

Of the 27 million surgeries performed in the United States each year, a reported 2.6% result in a surgical site infection (SSI), and *Staphylococci* species are commonly the culprit. Alternative therapies, such as nitric oxide (NO)-releasing biomaterials, are being developed to address this issue. NO is a potent antimicrobial agent with several modes of action, including oxidative and nitrosative damage, disruption of bacterial membranes, and dispersion of biofilms. For targeted antibacterial effects, NO is delivered by exogenous donor molecules, like *S*-nitroso-*N*-acetylpenicillamine (SNAP). Herein, the impregnation of SNAP into poly(lactic-*co*-glycolic acid) (PLGA) for SSI prevention is reported for the first time. The NO-releasing PLGA copolymer is fabricated and characterized by donor molecule loading, leaching, and the amount remaining after ethylene oxide sterilization. The swelling ratio, water uptake, static water contact angle, and tensile strength are also investigated. Furthermore, its cytocompatibility is tested against 3T3 mouse fibroblast cells, and its antimicrobial efficacy is assessed against multiple *Staphylococci* strains. Overall, the NO-releasing PLGA copolymer holds promise as a suture material for eradicating surgical site infections caused by *Staphylococci* strains. SNAP impregnation affords robust antibacterial properties while maintaining the cytocompatibility and mechanical integrity.

## 1 Introduction

Surgical site infections (SSIs) arise when pathogenic bacteria colonize the portion of the body where surgery has taken place, resulting in redness, delayed healing, fever, pain, tenderness, warmth, and swelling. Of the 27 million surgeries performed in the United States each year, a reported 675,000 result in an SSI and account for roughly 20% of all hospital-acquired infections (HAIs).^1−3^ In low- and middle-income countries, SSIs are the leading cause of HAIs.^4^ The high prevalence of SSIs presents a significant burden on the healthcare industry because of increased patient morbidity and mortality, increased duration of hospital stay, and increased financial liability.^5^ A solution is enhanced antimicrobial medical devices, such as surgical sutures, to kill bacteria in the surrounding wound environment and prevent the adherence of bacteria to the material itself. For a controlled dosing of antibiotics and antiseptic agents against SSI-related pathogens, sutures are commonly coated with triclosan (polychloro phenoxy phenol),^6−9^ chlorohexidine,^10−13^ or silver nanoparticles.^14−16^ Triclosan has been commercially used as an antibacterial suture coating since its FDA approval in 2002.^8,9^ Nevertheless, antibiotic- and antiseptic-coated sutures are threatened by the onset of antimicrobial resistance (AR). *Staphylococci* strains are the most common culprit in SSIs, and some strains have already developed resistance to conventional antibiotics—for example, Methicillin-resistant *Staphylococcus aureus* (MRSA) and Vancomycin-resistant *Staphylococcus aureus* (VRSA).^17−19^

Alternative therapies to combat AR, such as nitric oxide (NO)-releasing biomaterials, are being developed. NO is a naturally occurring gaseous molecule produced by macrophages to aid in killing Gram-positive and Gram-negative bacterial cells.^20^ The molecule has potent antibacterial characteristics, with several modes of action, including (1) disruption of bacterial membranes, (2) oxidative and nitrosative damage toward bacterial DNA and proteins, and (3) dispersal of bacterial biofilms.^21−23^ NO has a short half-life (in the order of seconds) and the multimechanistic antibacterial effects occur rapidly, thus inhibiting resistance development. Due to this instability, donor compounds are needed to stabilize its release. In the biomaterials field, there are two common classes of NO-donor compounds: *S*-nitrosothiols (RSNOs) and *N*-diazeniumdiolates (NONOates).^24−26^ NO-releasing biomaterials are well-established in their antibacterial properties.^27−31^ Recently, RSNOs have gained popularity over NONOates due to their simplistic synthesis methods and steady release under physiological conditions.^26,32^*S*-nitroso-*N*-acetylpenicillamine (SNAP) is an RSNO compound whose NO release is catalyzed upon stimulation from light, heat, metal ions, and hydrolysis.^32^ NO donors can be further stabilized by incorporating them into various delivery platforms. Previous studies have shown that the addition of NO donors to poly(lactic-*co*-glycolic acid) (PLGA) micro- and nanosized particles leads to desirable pharmacokinetic properties—NO release is more stable when in solution^33−38^ and when functionalized to a surface.^39,40^ PLGA has also been used as an additive^41−43^ and a block copolymer component^44−47^ to extend NO release profiles considerably. In these instances, oil-in-water solvent evaporation and emulsification methods were used to prepare PLGA micro- and nanoparticles with NO, and solvent casting was used to create NO-releasing polymers. However, solvent swelling methods have not been used for incorporating NO into a postfabricated PLGA polymer material.

This manuscript proposes impregnating the NO-donor SNAP into the copolymer 10:90 PLGA to reduce SSIs due to *Staphylococci*. The PLGA copolymer formation used is 10% l-lactide: 90% glycolide to represent Ethicon’s VICRYL^TM^ (polyglactin 910) sutures, one example of a commercially available suture material.^48,49^ The ratio of l-lactide and glycolide was kept the same as that of commercially available sutures to mimic their physiochemical and mechanical material properties. We hypothesize that the solvent swelling methods used to impregnate SNAP will not affect the copolymer’s mechanical properties (maintaining suture integrity) and that the localized delivery of NO will reduce bacterial viability (reducing SSI prevalence). The SNAP loading, SNAP leaching, and NO release kinetics to optimize the concentration of the NO-donor in the swelling solution were characterized. The ideal NO-releasing sample type was then further characterized by water contact angle (WCA) to assess the effect of SNAP swelling on the surface of PLGA. Furthermore, tensile testing was conducted to evaluate the maintenance of mechanical strength, and ethylene oxide sterilization was performed to assess the ability to retain NO-release properties after sterilization. *In vitro* biological characterization consisted of cytocompatibility and antibacterial assays. Cytocompatibility testing was performed to ensure that the level of NO release from the ideal NO-releasing sample type did not elicit a cytotoxic response. Antibacterial assays were conducted against *Staphylococcus aureus* (*S. aureus*) and MRSA. The NO-releasing PLGA copolymer material proposed herein shows promise for combatting SSIs due to AR *Staphylococci* species.

## 2 Materials and Methods

### 2.1 Materials

10:90 PLGA (lot no. BB0306-163D, mol wt 100,000) was obtained from Bezwada Biomedical, LLC (Hillsborough, NJ). 1,1,1,3,3-Hexofluoro-2-propanol (HFIP) was purchased from Oakwood Chemical (Estill, SC). SNAP was purchased from PharmaBlock Sciences (Hatfield, PA). Ethylenediaminetetraacetic acid (EDTA) and tetrahydrofuran (THF) were purchased from Sigma-Aldrich (St. Louis, MO). Ethanol (EtOH) was purchased from VWR (Radnor, PA). Nonwoven all-purpose sponges and tegaderm were purchased from Fisher Scientific (Suwanee, GA). Deionized water for all aqueous solutions was obtained via an in-house distillation unit from Mettler Toledo (Columbus, OH). Nitrogen and oxygen gas cylinders were purchased from Airgas (Kennesaw, GA). Phosphate buffered saline (PBS), pH 7.4, had a final concentration of 138 mM sodium chloride, 2.7 mM potassium chloride, 10 mM sodium phosphate, and 100 μM EDTA.

For biological studies, mouse 3T3 fibroblast (ATCC 1658) cells were purchased from American Type Culture Collection (Manassas, VA). Dulbecco’s modified eagle medium (DMEM) and fetal bovine serum (FBS) were purchased from VWR (Atlanta, GA). *Staphylococcus aureus* (ATCC 6538) and Methicillin-resistant *Staphylococcus aureus* (ATCC BAA 041) were purchased from American Type Culture Collection (Manassas, VA). Tryptic soy and Mueller–Hinton broths and agars were purchased from Sigma-Aldrich (St. Louis, MO).

### 2.2 Material Fabrication

The chosen 10:90 PLGA formulation consists of a ratio of 10% l-lactide to 90% glycolide, which is equivalent to the common commercially used suture material polyglactin 910. Per the manufacturer, the purchased PLGA copolymer has an inherent viscosity of 1.84 dL g^–1^ at 0.1% in HFIP at 30 °C, a molecular weight of roughly 100,000 g mol^–1^, end group types consisting of ester and hydroxyl groups, and an *in vivo* adsorption time of 90 days. To create samples, 10:90 PLGA was dissolved in HFIP at 10 wt % overnight while stirring at room temperature (RT). The resulting solution was cast in glass Petri dishes and dried for 24 h. Punches were then made from the parent film to create 8 mm diameter samples, and the punches were stored at −20 °C with a desiccant for future use. For SNAP impregnation into the PLGA films, 25, 50, and 75 mg mL^–1^ solutions of SNAP in EtOH were prepared. PLGA samples were submerged in each of the SNAP solutions for 24 h on a rocker under dark conditions at RT. Samples were then removed from the swelling solution and allowed to air-dry in the dark at RT. After 24 h, the samples were sonicated for 10 s in DI water to remove any residual SNAP crystals from the surface. The samples were then dried with a kimwipe and placed in a desiccator for 24 h in the dark at RT. After, the SNAP-impregnated PLGA samples were stored at −20 °C with desiccant until use.

Presumably, changing the monomeric ratio of l-lactide to glycolide will affect the inherent viscosity and degradation rate, which may substantially affect NO release properties. However, the investigation of different monomer ratios is outside this manuscript’s objective. The scope of this article is to determine the optimum SNAP concentration for the novel solvent swelling of 10:90 PLGA for biomedical applications, like surgical sutures.

### 2.3 Material Characterization

#### 2.3.1 Swelling Ratio and Water Uptake

The swelling ratio of unmodified PLGA in EtOH was measured. First, the samples were dried at 80 °C for 1 h and weighed. The samples were then submerged in EtOH for 24 h at RT. The samples were then quickly blotted dry with a kimwipe and weighed. For the water uptake of modified PLGA, the samples were also dried at 80 °C for 1 h and weighed. Then, the samples were submerged in DI water for 24 h at RT. The samples were dried and weighed. The difference between the wet and dry weights for each sample was calculated, and the values were normalized by the dry weight. Results are reported as weight percent (wt %).

#### 2.3.2 SNAP Loading

The amount of SNAP impregnated into the PLGA samples via solvent swelling was assessed to compute the amount of SNAP loaded into the copolymer matrix during synthesis. Fabricated samples were placed in THF for 4 h to extract SNAP into the solution phase. The amount of SNAP present in the THF solution was measured via an Agilent Cary 60 UV–vis spectrophotometer (Santa Clara, CA) at 340 nm, corresponding to the *S*-nitrosothiol bond peak on the SNAP molecule (molar absorptivity of SNAP in THF is 0.40 mL mg^–1^ mm^–1^). Blank THF was used as the background. Additionally, the average absorbance (abs) of PLGA samples soaked in THF overnight versus blank THF was subtracted to correct the baseline for any PLGA noise. Corrected values were compared to a standard curve of known concentrations of SNAP in THF to quantify the amount of SNAP impregnated into the polymer matrix. All samples were normalized by weight. Results are reported as weight percent (wt %) (eq 1).
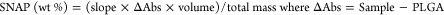
1

#### 2.3.3 SNAP Leaching

SNAP leaching from SNAP-impregnated PLGA samples was measured after a 12 h incubation period. Samples were placed in 0.01 M PBS containing 100 μM EDTA and incubated at 37 °C. Leachates were measured at 340 nm via UV–vis (the molar absorptivity of SNAP in PBS containing 100 μM EDTA is 0.48 mL mg^–1^ mm^–1^). Pure PBS containing EDTA was used as a blank control. Similarly, the average absorbance of PLGA samples soaked in PBS containing EDTA for 12 h was measured and subtracted to correct the baseline. Corrected values were compared with a standard curve of known concentrations of SNAP in PBS containing EDTA to quantify the amount of SNAP release present. All samples were normalized by weight, and the results are reported as weight percent (wt %)(eq 1).

#### 2.3.4 NO Release

NO release from NOrel-PLGA samples was measured via a Sievers Chemiluminescence Nitric Oxide Analyzer 280i (NOA) (Boulder, CO) in 0.01 M PBS containing 100 μM EDTA at 37 °C under dark conditions. A nitrogen bubbler carried any NO emitted by the samples into the NOA reaction chamber. In the NOA, oxygen is fed into an ozone generator, whereby ozone is passed into a reaction chamber with the NO sample gas. The NO reacts with ozone, forming nitrogen dioxide in an excited state (NO_2_*) (eq 2). The relaxation of NO_2_* leads to the emission of a photon, which passes through a photomultiplier tube to a detector, enabling NO readings in the ppb range. A calibration constant (mol ppb^–1^ min^–1^) enables the conversion of the ppb readings to surface flux over time (mol cm^–2^ s^–1^).
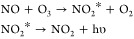
2

NOrel-PLGA samples were tested under moist and wet conditions. For wet conditions, samples were submerged in 0.01 M PBS containing an EDTA buffer solution (pH 7.4) inside an amber vial. For moist conditions, samples were wrapped in nonwoven gauze sponges dampened with the buffer solution; the gauze with the sample was then wrapped in Tegaderm to prevent buffer evaporation. The PBS buffer solution was supplemented with EDTA to prevent any trace metal ions from catalyzing the release of NO from the sample surface. For wet and moist setups, the samples were placed in a vial and submerged in a 37 °C water bath to keep the temperature constant. Stabilized NO flux values were analyzed at 0, 12, and 24 h.

#### 2.3.5 Static Water Contact Angle

An Ossila Contact Angle Goniometer (Sheffield, UK) was used to investigate the static contact angles of DI water on unmodified PLGA and the optimal NOrel-PLGA samples. One at a time, DI water droplets (5 μL) were lightly placed onto a film sample and allowed to settle in their static state. Once static, a picture of the droplet on the sample was taken, and the Ossila Contact Angle Software (v3.0.3.0) was used to determine the contact angle.

#### 2.3.6 Tensile Testing

Tensile testing was performed following ASTM D1708–18 standards with a slight modification. A Mark-10 Force and Tensile Measurement system (Copiague, NY) was used to determine the ultimate tensile strength (UTS) of unmodified PLGA and optimal NOrel-PLGA samples under dry, moist, and wet conditions. Films were prepared in 1 × 3 cm rectangular shapes. Samples were clamped in the Mark-10 system and subjected to increased load until breaking. The speed of the machine was 1 in. per minute. The gauge area of the samples was used to normalize the load at break measurement, and then UTS was calculated.

#### 2.3.7 Ethylene Oxide Sterilization

Optimal NOrel-PLGA samples were sterilized with ethylene oxide via an Andersen AN74i Anprolene Gas Sterilizer (Haw River, NC). Samples were inserted into a crosstex duocheck bag with indicators of a successful cycle. The samples were then placed into a liner bag containing an AN1071 humidi chip and an anprolene sterilizing gas ampule (17.6 g of ethylene oxide per ampule) and subsequently placed into the sterilizer system. The liner bag was then vacuum sealed, the ampule was broken, and the sterilizer system was shut to perform the 24-h cycle. After completion, samples were analyzed for the SNAP remaining via SNAP loading, as discussed previously in Section 2.3.2. Results are reported as a percent remaining, with fresh, unsterilized samples considered to be 100% (eq 3).
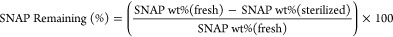
3

### 2.4 *In Vitro* Cytotoxicity Evaluation

To assess the cytotoxicity of unmodified PLGA and optimal NOrel-PLGA samples, a cell viability assay against NIH 3T3 mouse fibroblast cells was performed following ISO 10993–5 standards for the biological evaluation of medical devices.^50^ Cells were cultured in a T-75 flask containing DMEM media with 10% FBS and 1% penicillin–streptomycin (complete DMEM) at 37 °C with 5% CO_2_. Once 80% confluency was reached, cells were transferred to 96-well plates at a seeding density of 1 × 10^5^ cells mL^–1^. Concurrently, unmodified PLGA and NOrel-PLGA samples were soaked in complete DMEM for 24 h to obtain leachate solutions. The leachates were transferred to the cells in the 96-well plates. Exposure of the leachate solutions to the 3T3 cells in complete DMEM lasted 24 h at 37 °C and 5% CO_2_. After exposure, the leachate solutions were replaced with complete DMEM containing 10% CCK-8 solution, and the 96-well plates were incubated for an additional 2 h to develop the formazan dye. The yellow-orange dye is the result of 2-(2-methoxy-4-nitrophenyl)-3-(4-nitrophenyl)-5-(2,4-disulfophenyl)-2*H*-tetrazolium monosodium salt (WST-8) being reduced by the dehydrogenase activity of viable cells. Formazan was detected with a BioTeck Cytation5 plate reader (Winooski, VT) at 450 nm, and the dye concentration is directly proportional to the number of viable cells. Therefore, results are reported as percent viability compared to that of untreated 3T3 fibroblast cells (eq 4).
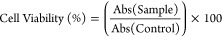
4

### 2.5 *In Vitro* Antibacterial Evaluation

*In vitro* antibacterial assays were carried out to quantify the ability of optimal NOrel-PLGA samples to reduce the concentration of planktonic and adhered bacteria. The 12-h exposure study followed a modified version of a prior protocol.^51^ Briefly, each strain was grown overnight at 37 °C and 150 rpm. *Staphylococcus aureus* (*S. aureus*) was inoculated in tryptic soy broth (TSB), while Methicillin-resistant *Staphylococcus aureus* (MRSA) was inoculated in Mueller–Hinton broth (MHB). Before using the inoculum, each culture was centrifuged at 4400 rpm for 7 min and suspended in PBS for a washing step. The culture was consequently centrifuged again and resuspended in PBS. The resuspended bacteria’s optical density (OD) was taken at 600 nm via UV–vis to ensure the bacteria culture was in the log phase of growth. Bacteria was diluted in PBS to 10^8^ colony-forming units (CFUs) per mL. Before exposure to bacteria, samples were sterilized by UV light for 15 min on each side.

#### 2.5.1 Planktonic and Adhered Bacteria in Viability Conditions

To quantify the number of viable planktonic bacteria under wet conditions, samples were placed in a 24-well plate, and 1 mL of prepared bacteria was pipetted into each well. The plate was then incubated at 37 °C and 150 rpm for 12 h. Solutions from the 24-well plate were serially diluted and next plated via an IUL Instruments Neutec Eddy Jet 2W spiral plater (Farmingdale, NY). The spiral plater was used in the log_mode_50 μL setting with 2 air purge cycles to plate a 3-fold dilution. For example, if the direct solution is used, then the 1× (direct), 10×, and 100× dilutions will be plated on the agar plates. *S. aureus* was plated on tryptic soy agar (TSA), while MRSA was plated on Mueller–Hinton agar (MHA). After the plates were incubated overnight, colony counting was performed via an IUL Instruments Neutec SphereFlash Colony Counter (Farmingdale, NY). Results are reported as a percent reduction by NOrel-PLGA or unmodified PLGA samples compared to untreated bacteria (eq 5).
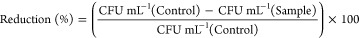
5

To quantify the number of viable adhered bacteria in wet conditions, the samples were removed from the well plate, gently washed with 1 mL of PBS, and placed in 15 mL centrifuge tubes containing 1 mL of PBS. Samples were homogenized for 60 s at 25,000 rpm to remove any adhered bacteria from the surface of the samples. Furthermore, the samples were vortexed for 1 min after homogenization. The solutions were then serially diluted and plated. The same plating and counting process was performed by utilizing a Neutec Eddy Jet 2W spiral plater and SphereFlash Colony Counter, respectively. Results are reported as a percent reduction by NOrel-PLGA samples compared to unmodified PLGA (eq 6).
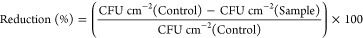
6

#### 2.5.2 Zone of Inhibition

To evaluate the ability of NOrel-PLGA to prevent bacterial growth in moist conditions, the relative zone of inhibition (ZOI) against unmodified PLGA was measured with modifications from previously published protocols.^52,53^*S. aureus* and MRSA strains were diluted to 10^8^ CFUs mL^–1^ and 50 μL of solution was spread on agar plates using sterile cotton swabs. Samples were gently pressed onto the agar plates, and 10 μL of sterile PBS was pipetted onto the samples. Sterile nonwoven gauze sponges were dampened with sterile PBS and placed in the lid of the agar plates. The plates were wrapped in parafilm and kept at 37 °C for 24 h, after which images of the agar plates were captured via a Neutec SphereFlash Colony Counter. Then, the ZOI was measured via digital calipers.

#### 2.5.3 Minimum Inhibitory Concentration (MIC) Testing

The MIC for SNAP against *S. aureus* and MRSA was determined using a broth microdilution assay with minor deviations.^54^ The bacterial inoculums were prepared as detailed in Section 2.5 except that the centrifuged bacteria was resuspended and diluted in media to roughly 10^8^ CFUs per mL. Meanwhile, stock solutions of SNAP (24 mM) in media were prepared at 2× the desired final concentration, and equal volumes of SNAP and bacteria solutions were added to the appropriate wells in a 96-well plate. Final SNAP concentrations ranged from 62.5 μM to 12 mM. The prepared plates were incubated at 37 °C in the dark for 24 h at 150 rpm. After 24 h, each well’s OD was measured at 600 nm using a Cytation5 plate reader (BioTek, Winooski, VT). Blanks for the media and each treatment were subtracted in analysis. The relative OD of the treated bacteria wells was normalized to untreated bacteria (eq 7).
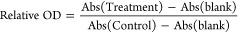
7

### 2.6 Statistical Analysis

All measured data are reported as a mean ± standard deviation (SD) with *n* ≥ 3. Statistical analysis was completed in GraphPad Prism Software v9.1 (San Diego, CA). As appropriate, unpaired *t* tests and one-way ANOVA with correction for multiple comparisons between means of each sample group using Tukey’s method were used to determine statistical significance. For antibacterial studies, analysis was performed on logarithmic calculations.

## 3 Results and Discussion

### 3.1 Characterization of Samples

#### 3.1.1 SNAP Loading

SSIs arise when pathogenic bacteria, most often *Staphylococci* strains,^17−19^ infect the surgical site, causing increased patient morbidity and mortality, duration of hospital stay, and financial liability.^5,8^ Additionally, SSI incidences account for nearly 20% of all HAIs.^4^ Moreover, the rise of antimicrobial resistance poses a significant challenge in managing SSIs, given the numerous strains of *Staphylococci*, such as MRSA, that are categorized as antimicrobial resistant. NO is an attractive alternative because it is a gaseous molecule with potent antimicrobial qualities and multiple modes of action, including (1) membrane disruption, (2) oxidative and nitrosative destruction, and (3) biofilm dispersal.^21−23^ SNAP is a NO-donor compound that can be readily incorporated into postfabricated medical-grade materials via solvent swelling.^55^ This work investigates, for the first time, the solvent swelling of SNAP into PLGA to render the copolymer NO-releasing and to demonstrate a novel approach for treating SSIs. The 10:90 PLGA copolymer films were cast and impregnated with SNAP, characterized regarding their NO release properties, evaluated for any change in physical and mechanical properties, and tested for antibacterial efficacy against multiple *Staphylococci* strains.

First, the ability to swell the PLGA copolymer was investigated to develop an optimal SNAP swelling system. It has already been demonstrated that several organic solvents can swell silicone rubber,^56^ and tetrahydrofuran (THF) is traditionally used for SNAP impregnation.^57−59^ However, PLGA is soluble in a wide variety of solvents, including THF,^60,61^ making it an inappropriate choice for swelling in this work. A better choice is ethanol (EtOH). PLGA does not dissolve in this solvent, with no significant weight change after submersion for 24 h, and it swells at a ratio of 2.19 ± 0.76 wt % (*n* > 5). Therefore, EtOH was chosen as the solvent for impregnating SNAP into the PLGA copolymer matrix.

**Figure 1:**
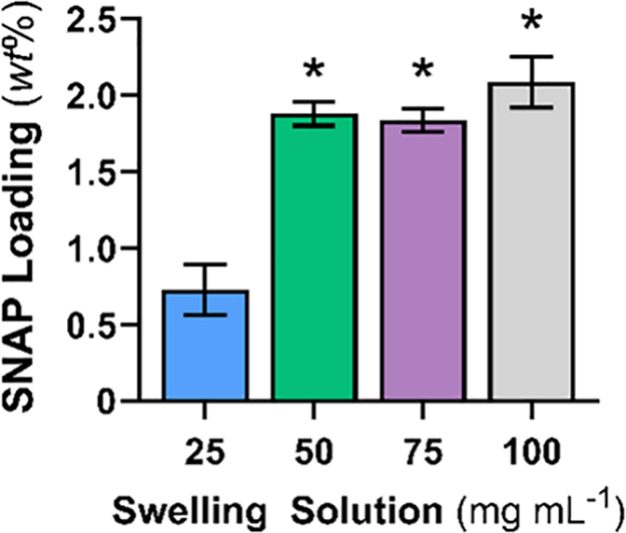
SNAP loading quantification into PLGA from 25, 50, 75, and 100 mg mL^–1^ swelling solution concentrations for 24 h. Values are represented as mean ± SD (*n* = 3) with * indicating statistical significance (*p* < 0.0001) against 25 mg mL^–1^ concentration.

SNAP was swollen into the PLGA matrix at various concentrations (25, 50, 75, and 100 mg of SNAP per mL of EtOH), and the samples were assessed for their ability to retain SNAP from the swelling solutions (Figure 1). To measure the amount of SNAP loaded, samples were submerged in THF for 4 h in the dark to extract the impregnated SNAP. The absorbance of the solution was measured at 340 nm, corresponding to the *S*-nitrosothiol bond peak on the SNAP molecule (Figure S1), and compared to a concentration calibration curve. Values are reported as wt % (see Supporting Information, Table S1). As expected, swelling with 50 mg mL^–1^ SNAP resulted in a higher loading than 25 mg mL^–1^. However, 75 mg mL^–1^ and 100 mg mL^–1^ did not yield significantly more SNAP loading than 50 mg mL^–1^. This finding demonstrates that SNAP impregnation into PLGA plateaus at 50 mg mL^–1^ SNAP in EtOH. Solvent swelling with 50 mg mL^–1^ in EtOH is desirable over higher 75 and 100 mg mL^–1^ concentrations. From a materials point of view, the plateau in SNAP loading is likely because SNAP has a specific solubility in the PLGA copolymer. At some point, the concentration in the solvent saturates the copolymer, and therefore, the material cannot absorb additional SNAP. It is safe to assume that 50 mg mL^–1^ saturated PLGA since increasing the solvent swelling solution past this value did not result in higher SNAP loading.

#### 3.1.2 SNAP Leaching

Due to plateaued SNAP loading, only 25 and 50 mg mL^–1^ samples were further characterized, beginning with SNAP leaching from the polymer matrix. To measure SNAP leaching, samples were submerged in PBS containing EDTA, incubated at 37 °C, and the absorbance of the solution was read at 340 nm after 12 h. The results show that more SNAP leaching occurred from 50 mg mL^–1^ samples than from 25 mg mL^–1^ samples (see Supporting Information, Table S1). For both sample types, roughly 60–70% of SNAP incorporated leached into the surrounding solution. This high level of leaching was expected since the copolymer consists of 90% glycolide, which is hydrophilic (copolymer hydrophilicity confirmed via contact angle, see Section 3.1.4) and has reasonable water uptake of 5.32 ± 0.22 wt % (*n* > 5). As water infiltrates the material, SNAP is transferred out of the matrix and into the surrounding solution. Even though the levels of SNAP leaching are high, they are not cytotoxic (see Section 3.2.1), and the leachate kills significant planktonic bacterial levels in the surrounding environment (see Section 3.2.2).

#### 3.1.3 NO Release

**Figure 2:**
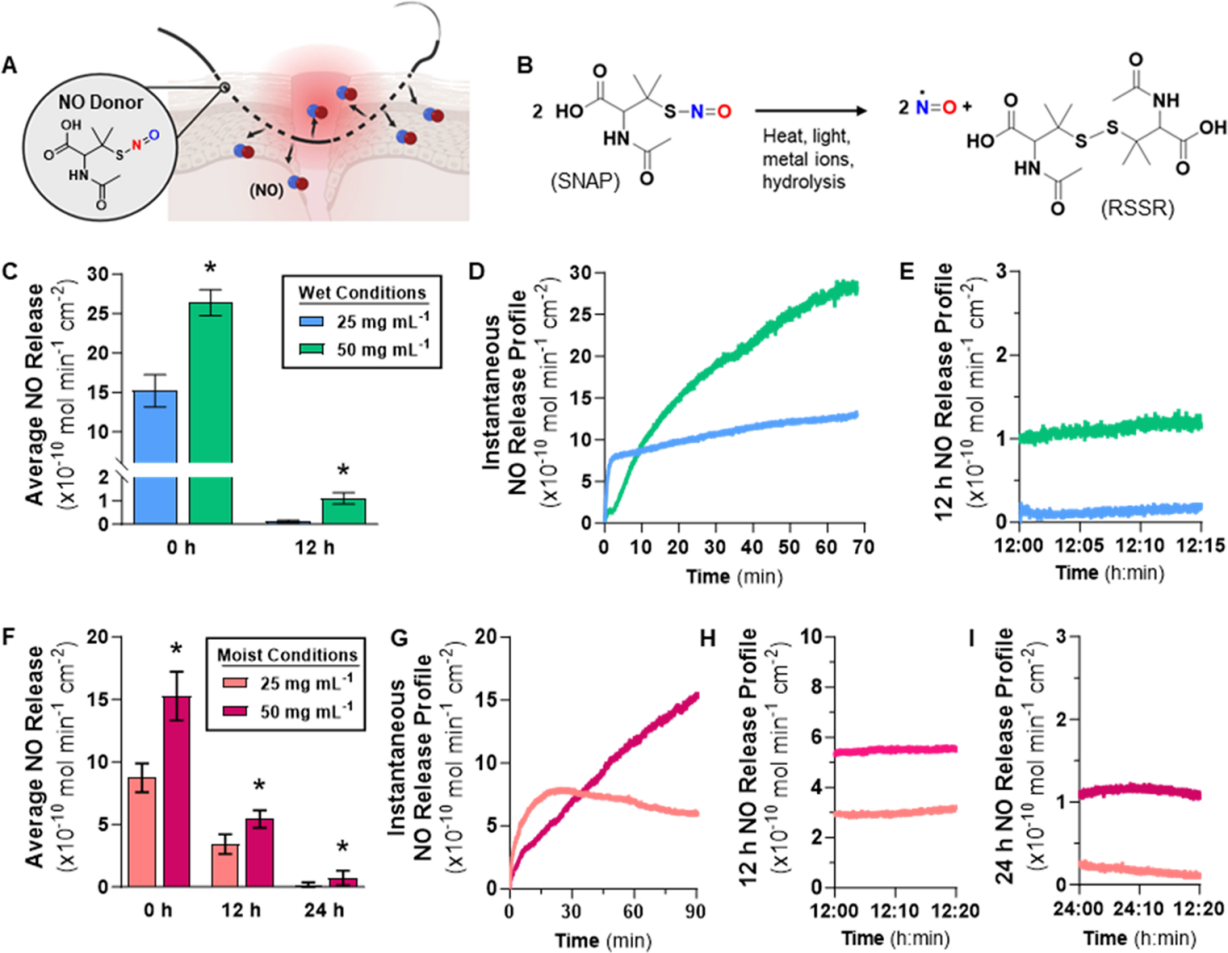
(A) NO release characterization studies of NOrel-PLGA. (B) NO is released upon the degradation of SNAP by heat, light, and metal ions. (C) For wet conditions, average NO release at 0 and 12 h time points, and representative instantaneous NO release profiles at (D) 0 h and (E) 12 h. (F) For moist conditions, average NO release at 0, 12, and 24 h time points, and representative instantaneous NO release profiles at (G) 0 h, (H) 12 h, and (I) 24 h. Values are represented as mean ± SD (*n* = 3) with * indicating statistical significance (*p* < 0.05) against 25 mg mL ^–1^ samples.

Next, NO release from the SNAP-impregnated samples (Figure 2A) was measured by using a gold-standard NOA instrument. Both wet and moist environments were simulated by submerging the samples in PBS containing EDTA or placing samples in gauze previously dipped in PBS containing EDTA. Under these conditions, NO release from SNAP occurs due to heat- and acid-catalyzed hydrolysis (Figure 2B). The amber chamber protects samples from light, and EDTA chelates any metal ions. The results presented here are consistent with previous literature demonstrating that although SNAP impregnation is a simple fabrication method, it often results in a high initial release of NO that tapers down over time.^55,62−65^ The release of NO from SNAP-impregnated PLGA kills bacteria adhered to the surface of the copolymer material (see Section 3.2.2).

In wet conditions, samples swelled at 50 mg mL^–1^ give a significantly higher NO release than that at 25 mg mL^–1^ (Figure 2C). At physiological conditions, the 25 mg mL^–1^ samples quickly reached equilibrium and exhibited a stable release of NO over a 1 h period; on the other hand, the 50 mg mL^–1^ samples stabilized after 1 h (Figure 2D). After 12 h in wet conditions, the samples swelled at 50 mg mL^–1^ give an NO release profile statistically greater than 25 mg mL^–1^ samples (Figure 2E). Higher NO release is likely due to more SNAP loading and overall SNAP remaining in the copolymer matrix. Since the SNAP reservoir is finite and PLGA is hydrophilic, fabricated samples are exhausted of NO before 24 h. To continue investigating the trend in NO release from SNAP-impregnated PLGA, samples were also evaluated under moist conditions (Figure 2F–I). In moist conditions, samples swelled at 50 mg mL^–1^ give an initial NO release higher than 25 mg mL^–1^. Compared to wet conditions, the release of NO in a moist environment is more controlled, and the initial release of NO is much lower. Consequently, the moist 12 h release profiles exhibit lower flux values than wet conditions. The finite SNAP reservoir is depleted less quickly, and the samples continue to release NO at 24 h. Subsequently, the 50 mg mL^–1^ samples were determined to be the optimal sample type due to maximum SNAP loading and statistically higher NO release levels than those of 25 mg mL^–1^ samples for both wet and moist conditions. Moving forward, unmodified PLGA control samples (Unmod. PLGA) and 50 mg mL^–1^ of SNAP in EtOH samples (NOrel-PLGA) are used for physical characterizations and *in vitro* biological evaluations.

#### 3.1.4 Static Water Contact Angle

**Figure 3:**
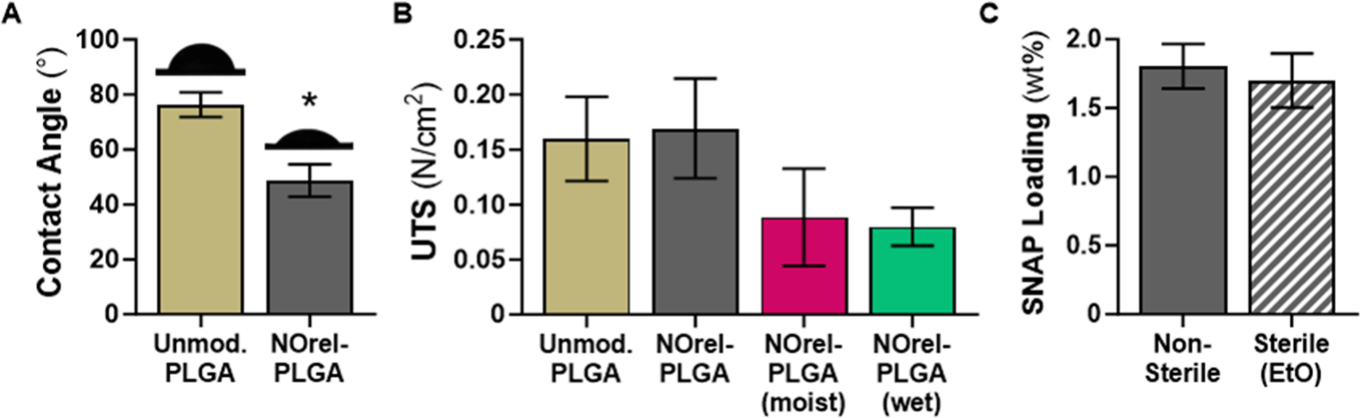
(A) Contact angle measurements and representative images. (B) Tensile testing in dry, moist, and wet conditions. (C) Ethylene oxide sterilization. Values are represented as mean ± SD (*n* > 3) with * indicating statistical significance (*p* < 0.0001) against unmodified PLGA.

Water contact angle (WCA) measurements were evaluated to confirm the hydrophilic properties of unmodified PLGA along with the optimal NOrel-PLGA samples (Figure 3A). Unmodified PLGA films demonstrated a hydrophilic WCA. The presence of SNAP significantly decreased the WCA, which is consistent with previous literature.^62^ SNAP causes a decrease in the WCA due to nitrogen and oxygen on the copolymeric surface, resulting in increased hydrogen bonding with the water. These hydrophilic properties led to high SNAP leaching (see earlier discussion in Section 3.1.2) from the PLGA copolymer matrix, which benefits planktonic antibacterial effects.

#### 3.1.5 Tensile Testing

For SNAP impregnation of the 10:90 PLGA to be a commercially viable option for rendering PLGA sutures NO-releasing and preventing SSIs, the mechanical integrity of the material cannot be affected. Consequently, tensile testing was conducted to evaluate the ultimate tensile strength (UTS) of unmodified PLGA and the optimal NOrel-PLGA samples (Figure 3B). There is no statistical significance in UTS between unmodified PLGA and optimal NOrel-PLGA in dry conditions. When NOrel-PLGA is exposed to moist conditions for 24 h and wet conditions for 12 h, there is a statistically insignificant decrease in UTS. Per the manufacturer, 10:90 PLGA degrades *in vivo* within 90 days. Therefore, the effect of EtOH and SNAP on the mechanical properties of the copolymer is presumed to be negligible. Subsequently, if commercially available 10:90 PLGA-based sutures, such as polyglactin 910, are modified to be NO-releasing via SNAP in EtOH solvent swelling methodology, mechanical properties such as elasticity and tensile strength will not be affected.

#### 3.1.6 Ethylene Oxide Sterilization

Likewise, NO-releasing PLGA sutures must retain loaded SNAP after commercial sterilization processes. Ethylene oxide gas is a standard method for sterilization of heat- and moisture-sensitive materials. After ethylene oxide (EtO) treatment, the sterile NOrel-PLGA samples retained 94.25 ± 10.89% of the SNAP loaded initially into the copolymer matrix (Figure 3C). Previous work corroborates that a trivial amount of SNAP is lost during ethylene oxide sterilization.^66^ The ability of this material to be sterilized without SNAP degradation is essential if NOrel-PLGA is to be used in a hospital setting to prevent SSIs.

### 3.2 *In Vitro* Biological Characterization

#### 3.2.1 Cytotoxicity Evaluation

**Figure 4:**
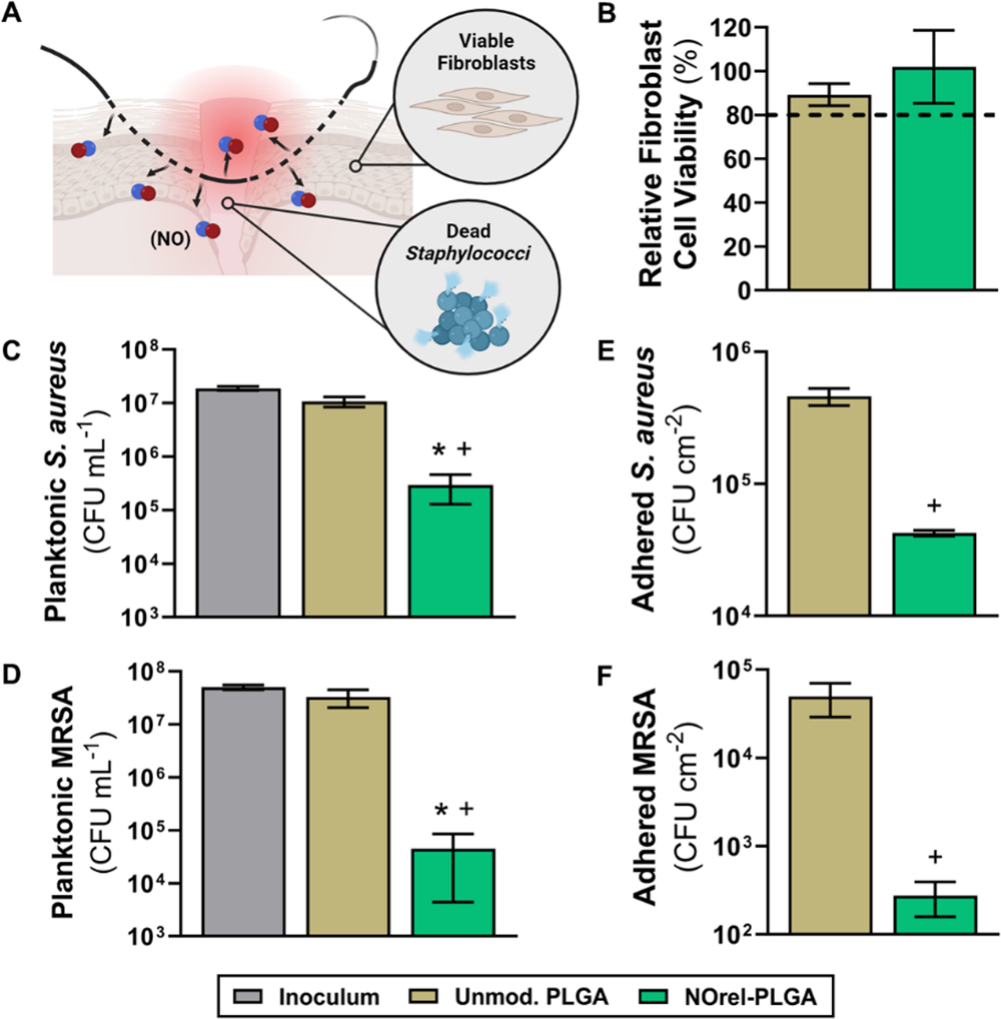
(A) Biological studies of unmodified PLGA and NOrel-PLGA. (B) The cytotoxicity measurements were normalized to those of untreated 3T3 mouse fibroblast cells. Bacterial reduction of the NOrel-PLGA samples is shown against two *Staphylococci* strains after 12 h. Planktonic antibacterial data for (C) *S. aureus* and (D) MRSA. Adhered antibacterial data for (E) *S. aureus* and (F) MRSA. Values are represented as mean ± SD (*n* ≥ 3) with * (*p* < 0.001) and + (*p* < 0.0001) indicating statistical significance against unmodified PLGA and untreated bacteria, respectively.

For SNAP-impregnated PLGA to be deemed appropriate for clinical use, the material must maintain cytocompatibility while having potent antibacterial effects (Figure 4A). Therefore, unmodified PLGA and optimal NOrel-PLGA were evaluated against 3T3 mouse fibroblast cells following ISO 10993–5 standards for the biological evaluation of medical devices.^50^ No cytotoxic response was observed, as the percent viability of each sample group relative to untreated cells remained above 80% (Figure 4B). There is no statistical significance between unmodified PLGA and NOrel-PLGA viability. Overall, SNAP leaching and NO release byproducts (i.e., peroxynitrite, nitrite, and disulfide dimer) levels are not cytotoxic, further supporting the use of this cytocompatible material for *in vitro* bacteria studies. These results are further consistent with prior reports of NO-releasing materials, wherein direct contact testing of materials lead to no significant cytotoxic effects at NO release rates comparable to the present PLGA formulations.^67,68^

#### 3.2.2 Antibacterial Evaluation

Surgical sites often become colonized with bacteria, most commonly *Staphylococci* strains,^17−19^ leading to redness, delayed healing, tenderness, warmth, and swelling. NO-releasing biomaterials prevent bacterial adhesion and kill planktonic bacteria^55,63,64,69,70^ through several mechanisms, including DNA cleavage, lipid peroxidation, and nitrosative and oxidative stress.^21−23^ To assess the antibacterial efficacy of the optimal NOrel-PLGA samples, *in vitro* antibacterial assays were conducted under moist and wet conditions. Zone of inhibition testing was performed to demonstrate the antibacterial properties of NO released from the samples in moist conditions. For wet conditions, samples were submerged in a bacteria solution to quantify the viable planktonic and adhered bacteria. Studies were completed with two *Staphylococci* strains: *S. aureus* and MRSA.

As depicted by the ZOI results, the NO diffused from the NOrel-PLGA samples hinders the growth of bacteria (see Supporting Information, Figure S2). The presence of SNAP limits the growth and development of *Staphylococci* bacteria in a moist environment. Zone analysis reveals that in moist conditions the strain of MRSA used in this work is more susceptible to NO diffusion than *S. aureus* (Table 1). When evaluating NOrel-PLGA in wet conditions, the SNAP leaching and NO release levels significantly reduced viable planktonic and adhered bacteria (see Supporting Information, Tables S2 and S3, respectively). Compared to the enumerated inoculum, the NO-releasing PLGA demonstrates a significant planktonic reduction: 1.80-log against *S. aureus* and 3.05-log against MRSA (Figure 4C and 4D, respectively). The material’s hydrophilicity encourages water uptake and, consequently, SNAP leaching into the surrounding environment. The leached SNAP facilitates the substantial killing of planktonic bacteria. Furthermore, the NO-releasing material demonstrates a statistically significant decrease in viable adhered bacteria: 1.03-log versus *S. aureus* and 2.25-log versus MRSA (Figure 4E and Figure 4F, respectively). The increased susceptibility of MRSA to NO-release from SNAP is supported by minimum inhibitory concentration (MIC) testing (see Supporting Information in Figure S3). Analysis revealed that SNAP prevents visible MRSA growth at a lower concentration than that for *S. aureus*, indicating the MRSA strain used herein is more susceptible to NO.

**Table 1:** Zones of Inhibition (ZOI) for Unmodified PLGA and NOrel-PLGA against *S. aureus* and MRSA^a^

material	*S. aureus* ZOI (mm)	MRSA ZOI (mm)
unmodified PLGA	no zone	no zone
NOrel-PLGA	15.67 ± 1.53	21.50 ± 2.78

a Values are represented as the mean ± SD (*n* = 3).

The antibacterial results suggest that SNAP-impregnated 10:90 PLGA is an appropriate antibacterial suture material for preventing infections at surgical sites. Solvent swelling methodology results in anti-*Staphylococci* effects under physiological conditions relevant to suture usage. In detail, NO-donor leachate levels are not cytotoxic and provide greater than a 3-log reduction against antimicrobial resistant bacteria. NOrel-PLGA’s strong antibacterial and cytocompatible properties suggest that it can prevent SSIs and be an alternative to antibiotic prophylaxis. Although CDC guidelines emphasize good surgical techniques to prevent SSIs, antimicrobial prophylaxis is commonly used, and the first dose should be given within 1–2 h after surgery.^3,71^ A delay in preventative treatment directly leads to increased SSI risk, but using antibiotics in this manner unavoidably increases antimicrobial resistance.^71^ NOrel-PLGA maintains a physiologically relevant NO release profile for 12 h, covering the time frame when preventative antibiotic and antiseptic doses are given. Therefore, NO-releasing PLGA sutures provide an appealing alternative to antibiotic prophylaxis to prevent SSIs.

## 4 Conclusion

The annual cost of HAIs is $9.8 billion US dollars, with SSIs being the most common.^72^ This article presents NO-releasing PLGA as an antibacterial surgical suture alternative to prevent SSIs due to *Staphylococci* bacteria. The optimal material was achieved by swelling the copolymer with 50 mg mL^–1^ SNAP in EtOH. At this concentration, NO-donor loading plateaued, and despite a high initial release of NO, physiologically relevant levels were maintained after 12 h under wet and 24 h under moist conditions. More than half of the donor molecule was released in the 12-h period due to the copolymer’s hydrophilic nature. Tensile testing revealed that nitric oxide donor impregnation did not impact the mechanical properties, and ethylene oxide sterilization did not affect the amount of donor impregnated in the samples (∼4% difference before and after sterilization). As demonstrated by *in vitro* biological evaluation, NO-releasing samples do not elicit cytotoxic responses from 3T3 mouse fibroblast cells while maintaining the ability to kill bacteria in the environment and adhered to the material surface. Surgical sites are most often colonized by *Staphylococci* strains,^17−19^ and accordingly, samples were tested against two *Staphylococci* strains, leading to a 1.80- and 3.05-log reduction against planktonic *S. aureus* and MRSA, respectively. Additionally, samples resulted in 1.03- and 2.25-log reductions in adhered *S. aureus* and MRSA, respectively. An inhibition zone against these two *Staphylococci* strains was also observed for the NOrel-PLGA samples.

Overall, our findings demonstrate the applicability of SNAP-impregnated PLGA for biomedical applications such as sutures, therefore reducing the burden SSIs and antibiotic prophylatic usage place on the healthcare industry. The results presented herein recommend further investigation of PLGA-based materials for nitric oxide-releasing applications, more specifically, the effect of different monomeric ratios of l-lactide and glycolide on nitric oxide donor retention, nitric oxide release, mechanical properties, and overall antibacterial efficacy.

## Data Availability

Data will be made available on request.

## Supporting Information Available

NOrel PLGA suture supplemental: Experimental data for characterization (UV–vis spectra of SNAP in THF, SNAP loading and leaching) and biological evaluation (ZOI images, planktonic and adhered viability) (PDF)
