# Supplementary material for: Preventing Staphylococci Surgical Site Infections with a Nitric Oxide-Releasing Poly(lactic acid-co-glycolic acid) Suture Material
Source: ACS Appl Bio Mater. 2024 Apr 23;7(5):3086–95. doi: 10.1021/acsabm.4c00128 (PMC11110049; doi:10.1021/acsabm.4c00128)
Supplement: Supplementary file 1 — mt4c00128_si_001.pdf [file mt4c00128_si_001.pdf]

SUPPORTING INFORMATION

Preventing *Staphylococci* Surgical Site Infections with a Nitric Oxide-Releasing Poly(lactic acid-co-glycolic acid) Suture Material

Lauren Griffin [a], *Mark Richard Stephen Garren* [a], *Patrick Maffe* [a], *Sama Ghalei* [a], *Elizabeth J. Brisbois* [a], *and Hitesh Handa* [ab]*

[a] School of Chemical, Materials and Biomedical Engineering, College of Engineering, University of Georgia, Athens, GA, 30602, USA

[b] Department of Pharmaceutical and Biomedical Sciences, College of Pharmacy, University of Georgia, Athens, GA, 30602, USA

* Corresponding Author:

Dr. Hitesh Handa

Associate Professor

University of Georgia

302 East Campus Road

Athens, GA, 30602, USA

Telephone: (706) 542-8109

E-mail: hhanda@uga.edu

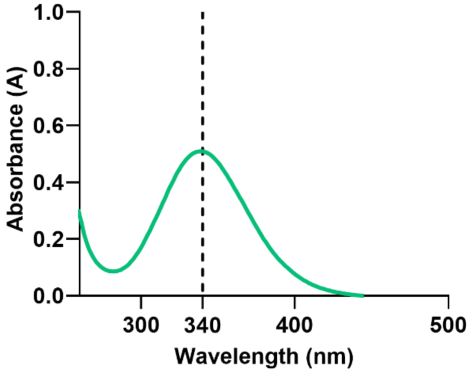

Figure S1. Representative UV-vis spectra of SNAP in THF, demonstrating the *S*-nitrosothiol bond peak on the SNAP molecule at 340 nm (molar absorptivity = 0.40 mL mg^{-1} mm^{-1}).

Table S1. SNAP loading and leaching for various swelling solution concentrations. Data is represented as mean ± SD (n = 3).

**Swelling Solution** (mg mL^{-1})	**SNAP Loading** (*wt*%)	**12 h SNAP Leaching** (*wt*%)
**25**	0.73 ± 0.17	0.52 ± 0.05
**50**	1.88 ± 0.08	1.17 ± 0.13
**75**	1.84 ± 0.07	n/a
**100**	2.09 ± 0.17	n/a

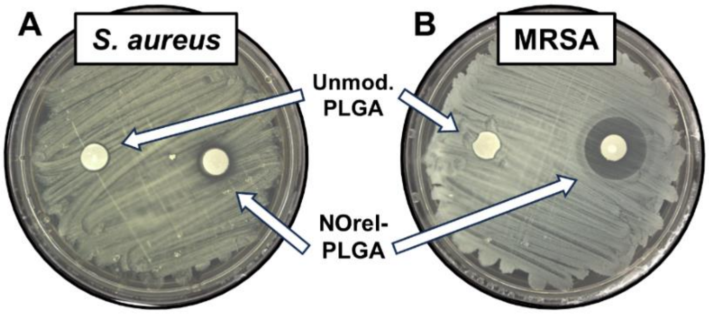

Figure S2. Representative images from zone of inhibition assay for (A) *S. aureus* and (B) MRSA. On each agar plate, unmodified PLGA is on the left, and NOrel-PLGA is on the right.

Table S2. Planktonic reduction against *S. aureus* and MRSA. Data is represented as mean ± SD (n = 3).

Sample	Unit	*S. aureus*	MRSA
**Inoculum** (Control)	CFU mL^{-1}	1.88 ± 0.16 ($x10^7$)	5.02 ± 0.54 ($x10^7$)
	Log reduction wrt. Control	n/a	n/a
	p value wrt. Control	n/a	n/a
**Unmod. PLGA**	CFU mL^{-1}	1.07 ± 0.23 ($x10^7$)	3.28 ± 1.20 ($x10^7$)
	Log reduction wrt. Control	0.25	0.18
	p value wrt. Control	0.3450	0.7766
**NOrel-PLGA**	CFU mL^{-1}	2.95 ± 1.60 ($x10^5$)	4.50 ± 4.06 ($x10^4$)
	Log reduction wrt. Control	1.80	3.05
	p value wrt. Control	< 0.0001	< 0.0001

Table S3. Adhered reduction against *S. aureus* and MRSA. Data is represented as mean ± SD (n = 3).

Sample	Unit	*S. aureus*	MRSA
**Unmod. PLGA**	CFU mL^{-1}	1.15 ± 0.17 ($x10^5$)	4.93 ± 2.04 ($x10^4$)
	Log reduction wrt. Control	n/a	n/a
	p value wrt. Control	n/a	n/a
**NOrel-PLGA**	CFU mL^{-1}	1.06 ± 0.05 ($x10^4$)	2.75 ± 1.18 ($x10^2$)
	Log reduction wrt. Control	1.03	2.25
	p value wrt. Control	< 0.0001	< 0.0001

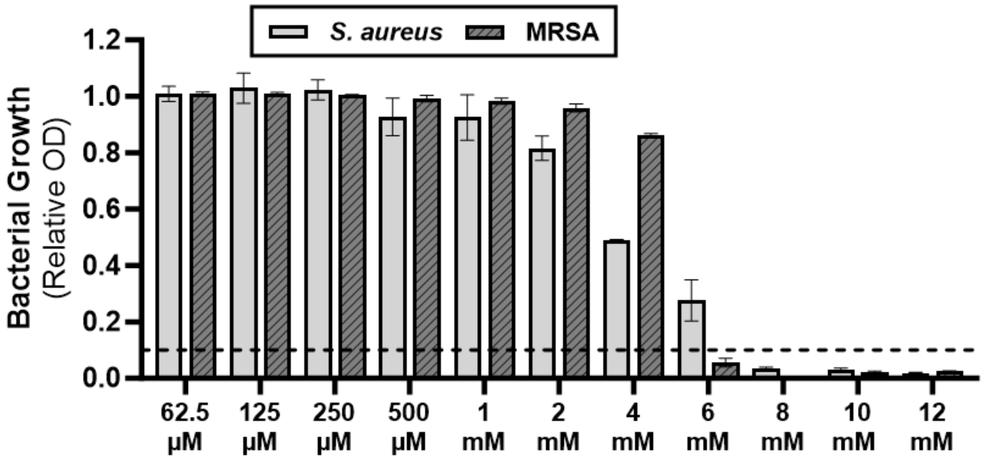

Figure S3. MIC results for SNAP against *S. aureus* and MRSA. Data is represented as mean ± SD (n = 3).
